# What If Pregnancy Is Not Seventh Heaven? The Influence of Specific Life Events during Pregnancy and Delivery on the Transition of Antenatal into Postpartum Anxiety and Depression

**DOI:** 10.3390/ijerph16162851

**Published:** 2019-08-09

**Authors:** Judith Aris-Meijer, Claudi Bockting, Ronald Stolk, Tjitte Verbeek, Chantal Beijers, Mariëlle van Pampus, Huibert Burger

**Affiliations:** 1 Department of Epidemiology, University of Groningen, University Medical Center Groningen, 9700 RB Groningen, The Netherlands; 2 Department of Clinical Psychology, University of Groningen, 3584 CS Groningen, The Netherlands; 3 Department of Clinical and Health Psychology, Utrecht University, 3512 JE Utrecht, The Netherlands; 4 Department of General Practice, University of Groningen, University Medical Center Groningen, 9700 RB Groningen, The Netherlands; 5 Interdisciplinary Center Psychopathology and Emotion Regulation, University of Groningen, University Medical Center Groningen, 9700 RB Groningen, The Netherlands; 6 Department of Obstetrics and Gynecology, OLVG, 1011 BM Amsterdam, The Netherlands

**Keywords:** antenatal anxiety and depression symptoms, postpartum anxiety and depression symptoms, mode of delivery, neonatal outcomes, life events

## Abstract

Introduction: Postpartum symptoms of anxiety and depression are known to have a negative impact on mother and child, and major life events constitute a major risk factor for these symptoms. We aimed to investigate to what extent specific life events during pregnancy, delivery complications, unfavorable obstetric outcomes, and antenatal levels of anxiety or depression symptoms were independently associated with postpartum levels of anxiety and depression symptoms. Methods: Within a prospective population-based cohort study (*n* = 3842) in The Netherlands, antenatal symptoms of anxiety or depression were measured at the end of the first trimester and at five months postpartum. Antenatal life events were assessed during the third trimester, and information on delivery and obstetric outcomes was obtained from midwives and gynecologists. Linear regression analyses were performed to quantify the associations. Results: Antenatal levels of both anxiety and depression symptoms were associated with postpartum levels of anxiety and depression symptoms. Life events related to health and sickness of self or loved ones, to the relation with the partner or conflicts with loved ones, or to work, finance, or housing problems were significantly associated with higher postpartum levels of anxiety symptoms (*p* < 0.001) and depression symptoms (*p* < 0.001) adjusted for antenatal levels. No statistically significant results were observed for pregnancy-related events, delivery complications, or unfavorable obstetric outcomes. Conclusions: Women with increased antenatal levels of anxiety or depression symptoms are at increased risk of elevated levels of both postpartum depression and anxiety symptoms. Experiencing life events during pregnancy that were not related to the pregnancy was associated with higher levels of anxiety and depression in the postpartum period, as opposed to pregnancy-related events, delivery complications, or unfavorable obstetric outcomes. These results suggest that events during pregnancy but not related to the pregnancy and birth are a highly important predictor for postpartum mental health.

## 1. Introduction

Reproductive age is a period of life in which women are vulnerable to the impact of symptoms of anxiety or depression. During and outside pregnancy alike, prevalence rates of these symptoms range from 8 to 15% [1,2,3,4,5,6,7,8,9,10,11,12]. Antenatal symptoms of anxiety or depression are the most important risk factor for the occurrence of these symptoms postpartum [3,13,14,15,16,17], which in turn has been associated with insecure mother–child attachment [18,19]. In addition, these symptoms during pregnancy have been associated with several obstetric adverse outcomes in the child, such as preterm birth and low birth weight [20,21,22], as well as emotional, cognitive, and behavioral problems [13,22,23,24,25,26].

Well-known risk factors for antenatal anxiety or depression are a history of anxiety or depression, low partner support, lower socioeconomic status, specific personality traits, and major life events [9,27,28]. Studies among pregnant women commonly classified recent major life events as general types of events during pregnancy [14,29], whereas other studies focused on pregnant women who were facing stress due to specific conditions, that is, obstetric complications [30,31]. A few population-based studies, however, have shown that specific pregnancy-related events are likely to increase symptoms of anxiety or depression during pregnancy [11,32,33].

Childbirth itself can also be considered a major life event, especially when the delivery is complicated, for example, when the baby is delivered by an emergency caesarean section or the baby is admitted to the neonatal intensive care unit. During the past decade, there is a growing but inconclusive body of literature on the associations between delivery complications and postpartum symptoms of anxiety and depression [34,35,36,37,38,39,40,41,42,43].

A few large population-based cohort studies (n > 5000) found that experiencing obstetric events during pregnancy or events that were related to the condition of the newborn (i.e., low birth weight, preterm delivery, congenital malformations, admission to the hospital) increased the risk of symptoms of depression in the postpartum period [33,44]. However, both studies underline the consensual idea that a history of symptoms of depression is the main risk factor for symptoms of depression at a later moment in time. Nevertheless, there is no answer yet to the question to what extent the combination of antenatal symptoms of anxiety or depression and specific life events during pregnancy or delivery is associated with postpartum symptoms of anxiety or depression.

Using a large population-based cohort study, we investigated to what extent specific life events during pregnancy, delivery complications, unfavorable obstetric outcomes, and antenatal levels of anxiety or depression symptoms independently contribute to the risk of these symptoms in the postpartum period and whether they interact.

## 2. Methods

### 2.1. Sample

Data were drawn from the prospective population-based Pregnancy, Anxiety and Depression (PAD) Study [11,12], which was designed to investigate symptoms of anxiety or depression and risk factors for antenatal and postpartum symptoms of anxiety or depression. Midwives and gynecologists of the collaborating primary obstetric care centers (*n* = 109) or hospitals (*n* = 7) invited pregnant women at the first or second visit to participate. It was impossible to establish how many women were actually invited to the study, as we had no insight into the total number of pregnant women who visited the primary obstetric care center or hospital for the first consult. The number of included women was however considerably lower than we expected based on the number of participating centers. A survey among participating midwives indicated that, for the vast majority, time pressure was the main reason to not hand out the invitations to all visiting women, and that women who were under suspicion of having symptoms of either anxiety or depression were not specifically invited. Therefore, we have no reason to believe that, with respect to characteristics relevant to the study, responders and non-responders differed in any considerable way. After written informed consent was obtained, women were requested to fill out online baseline questionnaires at the end of the first trimester, and online follow-up assessments at the end of the second and third trimesters of pregnancy, as well as at five months postpartum. The medical ethical board of the University Medical Center Groningen approved the PAD-based study.

Data used for the current study were collected between May 2010 and March 2015. Women who were at least four months postpartum were eligible to be included, as participants had the opportunity to fill out the follow-up questionnaire online between four and seven months postpartum. Exclusion criteria for the current sample were study withdrawal (*n* = 1669), not consenting to retrieve information from their midwives (*n* = 1669), no data on baseline and postpartum levels of anxiety and depression symptoms (*n* = 827), and no data on experienced life events during pregnancy or delivery (*n* = 1391). This resulted in a sample of 2450 women. Of these women, 2003 (81.8%) filled out the follow-up anxiety and depression questionnaires at five months postpartum.

For postpartum measures of anxiety and depression symptoms, responders were more often multiparous (*p* < 0.02). For postpartum measures of depression symptoms only, responders generally completed a higher education (*p* < 0.03). In addition, non-responders scored higher on antenatal measures of anxiety and depression compared to women who did respond to the postpartum follow-up questionnaire, although mean scores on antenatal anxiety and depression measures were below the prevailing cut-offs for both responders and non-responders. Women who did not respond to the follow-up questionnaire had experienced more general life events during pregnancy compared to women who did respond (*p* < 0.01), although the means differed less than one event for all categories. 

### 2.2. Measurements

Baseline levels of anxiety and depression symptoms measured at 12 weeks of estimated gestational age (range 5–19) and at five months postpartum (range 4–7) were analyzed. Life events during pregnancy were assessed during the third trimester. Maternal age and educational level were assessed at baseline. Educational attainment level was defined as the highest completed education and divided into four categories, namely, elementary or lower tracts of secondary education, higher tracts of secondary education, higher vocational education, and university education. Socioeconomic position was calculated as the equally weighted average of the educational attainment level of the respondent, her partner, and their total income.

Antenatal and postpartum symptoms of anxiety were measured using the six-item state measuring version of the Spielberger State-Trait Anxiety Inventory (STAI) [45]. Scores are on a scale from 20 to 80, with scores of ≥42 indicating an increased risk of anxiety [45].

To measure antenatal and postpartum symptoms of depression, we used the Dutch version of the ten-item Edinburgh Postnatal Depression Scale (EPDS) [46]. Scores range from 0 to 30. In line with Matthey et al. [47], we considered antenatal scores of 13 or above and postpartum scores of 10 or above to indicate risk of minor or major depression.

Data on life events that were encountered during pregnancy were collected using a 46 item questionnaire, developed in the Avon Longitudinal Study of Parents and Children (ALSPAC) [48]. We divided the events into four categories, namely, (A) work, finance, or housing problems, (B) partner relation or conflicts with loved ones, (C) health and sickness of self or loved ones, and (D) pregnancy-related. The first three comprise a total of 26 items on employment, illness or death of loved ones, and marital problems. The latter category includes seven items that are related to the current pregnancy, for example, undergoing tests on potential congenital anomalies of the fetus, being told that it is a twin pregnancy, finding out that the partner does not want to have the baby, or finding out that something that happened might be harmful for the fetus.

Information on mode of delivery and obstetric outcome was retrieved from the midwives’ or gynecologists’ reports. We defined delivery complications as instrumental vaginal delivery (i.e., forceps or vacuum extraction) or caesarean section (elective or emergency) relative to unassisted vaginal delivery.

Events that relate to the newborn were defined in terms of unfavorable obstetric outcomes and included preterm delivery (<37 weeks gestational age) and small for gestational age (i.e., >37 weeks gestational age but <2500 grams). Gestational age was derived from midwives’ and gynecologists’ reports. They calculated this age based on last menstrual period, and then confirmed it with an ultrasound.

### 2.3. Statistical Analyses

Descriptive statistics for demographic variables, number of life events, and levels of anxiety or depressive symptoms were calculated. To allow for valid comparison of effect sizes, we created z-scores for the antenatal symptoms of anxiety and depression. Subsequently, we performed a series of multivariable linear regression analyses with postpartum scores on STAI and EPDS to quantify the associations under study. The analyses quantified change in anxiety and depression symptom levels by adjusting postpartum levels for antenatal levels. In a separate analysis, potential confounders were added (i.e., socioeconomic position and nulliparity). Confounders were chosen based on their well-known association with the outcomes. To investigate associations with symptoms of anxiety or depression specifically, all analyses were additionally adjusted for depressive symptoms in the analysis of anxiety, and vice versa.

Lastly, we assessed whether associations of specific life events, delivery complications, and unfavorable obstetric outcomes of the newborn with postpartum symptoms of anxiety or depression were modified by antenatal anxiety or depression symptoms.

## 3. Results

### 3.1. General Descriptives

Mean levels for anxiety and depression were rather stable from the antenatal to the postpartum period (Table 1), although symptoms of depression significantly increased between the antenatal and postpartum period (mean difference = 0.36, *p* < 0.001).

### 3.2. Regression Analyses of Associations between Experienced Life Events and Postpartum Levels of Anxiety or Depression

Antenatal symptoms of anxiety and depression were statistically significantly associated with postpartum levels of anxiety (*p* < 0.001) (Table 2). Levels of postpartum anxiety increased more when women had higher levels of antenatal anxiety compared to antenatal depression; a score of one standard deviation higher on antenatal anxiety increased the postpartum anxiety scores, reaching 5.68 points, compared to the 2.78 point increase on postpartum levels of anxiety for one standard deviation higher on antenatal levels of depression. Likewise, levels of postpartum depression symptoms increased more when women had higher antenatal depression levels compared to anxiety (2.47 versus 0.57, respectively).

Neither the number of life events related to the pregnancy, nor the mode of delivery nor obstetric outcomes of the newborn were significantly associated with symptoms of anxiety and depression in the postpartum period. Adding potential confounders did not notably change the associations. When adjusting for postpartum levels of anxiety, all associations between life events and postpartum depression lost their statistical significance. For anxiety, this was only true for life events that were related to sickness and health of self or loved ones and life events related to the partner relation or a conflict with loved ones.

### 3.3. Moderation of the Associations between Experienced Life Events and Postpartum Levels of Anxiety or Depression by Antenatal Levels of Anxiety and Depression

The associations between number of life events and levels of postpartum anxiety symptoms were moderated by antenatal anxiety symptoms in the case of events related to sickness and health of self or loved ones (B 0.453, 95% CI 0.001–0.906, *p* = 0.049), and events related to work, finance, or housing problems (B 0.408, 95% CI 0.016–0.801, *p* = 0.041). However, after correcting for baseline levels of depression, none of the associations remained significant (B 0.158, 95% CI −0.124 to 0.763, *p* = 0.158 and B 0.242, 95% CI −0.145 to 0.629, *p* = 0.221, respectively).

The same trend was observed for the associations between antenatal life events related to work, finance, and housing problems and postpartum levels of depression symptoms (B 0.153, 95% CI 0.008–0.298, *p* = 0.039 versus B 0.144, 95%CI −0.001 to 0.288, *p* = 0.052).

## 4. Discussion

The present study confirmed previous research showing that antenatal symptoms of anxiety or depression during pregnancy are strongly associated with symptoms in the postpartum period. We showed that experiencing life events that were not related to the pregnancy, the mode of delivery, or the newborn were associated with elevated levels of anxiety and depression in the postpartum period. The association between postpartum levels of anxiety and events related to sickness and health of self or loved ones was found to be moderated by antenatal levels of anxiety.

Standardized effect sizes for levels of antenatal anxiety and depression were the highest of all predictor variables, indicating that antenatal symptomatology is the most important risk factor for having symptoms postpartum. This is in line with previous studies [3,13,14,15,17].

In our study, the categories of life events that related to health and sickness of self or loved ones, to the partner relation or a conflict with loved ones, or to work, finance, or housing problems (i.e., all categories that were not related to the pregnancy, delivery, or newborn) were associated with change in levels of anxiety and depression symptoms into the postpartum period. In the general population, housing problems have been found to increase feelings of stress [49]. Moreover, foreclosure induced a decline in mental health [50]. In addition, involuntary job loss and the past economic recession have been associated with higher suicidal rates [51,52]. However, this was not studied specifically in pregnant women and was especially found to be more prevalent in men.

Experiencing a major recent life event is widely considered to be an important risk factor for depression. We therefore hypothesized that childbirth could be considered a major life event when maternal and neonatal outcomes were complicated. Surprisingly, neither the events related to the pregnancy, to the delivery, nor to the condition of the newborn were associated with change in levels of anxiety and depression symptoms. For mode of delivery, there is an inconsistent pattern of findings in the literature, which may be due to methodological limitations such as small sample sizes or the fact of not distinguishing planned and unplanned caesareans [37]. Some recent studies did find associations between mode of delivery and depressed mood or comparable negative feelings [42,43], whereas several other studies underline our findings [34,35,39,40]. Another explanation for this inconsistency in findings may be that childbirth can be perceived as highly stressful and even result in post-traumatic stress syndrome (PTSD), even after experiencing a successful birth without any complications during childbirth or adverse maternal or neonatal outcomes [53]. We did not measure perception of the childbirth or coping strategies, but this could be an important factor for future research.

### Limitations and Strengths

Some limitations must be borne in mind. First, symptoms of anxiety and depression were based on self-report questionnaires. Although both are commonly used in the identification of symptoms and have shown to have good validity [45,46], no clinical diagnostic tools were used in the present study to establish the severity of symptoms. Second, life events were assessed using a retrospective self-report checklist, which may have been prone to recall bias through its potential link with symptoms at the time of the assessments [6]. Third, as the included midwifery practices and hospitals represent the general population in The Netherlands, and as we aimed to invite all pregnant women in their first trimester at the clinics, the included women could represent all women in The Netherlands, and by extension most Western countries. However, the vast majority of women in our sample had a high educational level (>60% have a higher vocational or university degree), which is not a solid representation of the general population. The obtained percentages of the main outcome measures, that is, postpartum anxiety and depression symptoms, were however in line with previous studies. Finally, although the total sample was large, we encountered a relatively high percentage of missing data on the outcome measures for the postpartum levels of depression and anxiety (18%). As the statistical power to demonstrate associations with a change in levels of anxiety and depression may thus be limited, these analyses should be considered exploratory.

However, a major strength of the present study is the large, prospective, population-based cohort (*n* = 2450). Additionally, to our knowledge, this study was the first to investigate the role of specific life events that are either more general or related to the pregnancy, delivery, or newborn, in relation to the specific symptoms of anxiety or depression in the postpartum period.

## 5. Conclusions

Our results indicate that the most important predictor for postpartum symptoms of anxiety or depression are elevated symptoms of antenatal anxiety or depression. Experiencing life events during pregnancy that were related to health and sickness of self or loved ones, to the partner relation or a conflict with loved ones, or to work, finance, or housing problems, was found to be associated with an increase in postpartum levels of anxiety and depression compared to antenatal levels. Midwives may play an important role in aiding women during pregnancy on coping with symptoms of anxiety or depression, and with the experience of such life events, in order to prevent postpartum levels of anxiety and depression from rising.

Experiencing life events during pregnancy that were related to the pregnancy, the mode of delivery, or the newborn was found not to be associated with an increase in postpartum levels of anxiety and depression. As perception of childbirth may be an important factor here, further research in this area should focus on quantifying complicated childbirth, and should take measures of resilience, coping strategies, and perception of childbirth into account.

## Figures and Tables

**Table 1:** Characteristics of women in the study.

Characteristics	All Women in the Study *n* = 3842	No Antenatal Symptoms *n* = 1948	Antenatal Anxiety and Depression Symptoms *n* = 85	Symptoms of Antenatal Anxiety, No Antenatal Depression *n* = 198	Symptoms of Antenatal Depression, No Antenatal Anxiety *n* = 7 *
Age at inclusion, mean (min–max)	30 (18–45)	30 (18–45)	30 (19–42)	31 (18–43)	32 (26–36)
Nulliparity, n (%)	1431/3682 (38.9%)	768/1915 (40.1%)	28/82 (34.1%)	78/198 (39.4%)	0/5
Educational level					
Elementary or lower tracts secondary	154/1879 (8.1%)	93/1360 (6.8%)	12/54 (22.2%)	18/119 (15.1%)	1/5
Higher tracts secondary	567/1879 (30.0%)	403/1360 (29.6%)	15/54 (27.8%)	41/119 (34.5%)	3/5
Higher vocational or university	1158/1879 (61.6%)	864/1360 (63.5)	27/54 (50.0%)	60/119 (50.4%)	1/5
No events related to the pregnancy, n (%)	1218/2449 (49.7%)	856/1685 (50.8%)	23/65 (35.4%)	65/147 (44.2%)	2/5
median (min–max)	1 (0–5)	0 (0–5)	1 (0–3)	1 (0–4)	1 (0–3)
No events related to health and sickness of self or loved ones, n (%)	1516/2407 (63.0%)	1080/1666 (64.8%)	29/64 (45.3%)	77/142 (54.2%)	2/5
median (min–max)	0 (0–8)	0 (0–8)	1 (0–6)	0 (0–4)	2 (0–5)
No events related to partner relation or conflicts with loved ones, n (%)	1909/2480 (77.0%)	1366/1705 (80.1)	29/65 (44.6%)	91/147 (61.9%)	1/5
median (min–max)	0 (0–4)	0 (0–4)	1 (0–4)	0 (0–4)	1 (0–2)
No events related to work, finance, or housing problems, n (%)	1151/2453 (46.9%)	845/1688 (50.1%)	11/64 (17.2%)	40/146 (27.4%)	1/5
median (min–max)	1 (0–9)	0 (0–8)	2 (0–8)	1 (0–7)	2 (0–6)
Mode of delivery, n (%)					
spontaneous vaginal delivery	1737/2343 (74.1%)	1190/1613 (73.8%)	41/59 (69.5%)	107/139 (77.0%)	4/5
vacuum or forceps extraction	244/2343 (10.4%)	173/1613 (10.7%)	6/59 (10.2%)	10/139 (7.2%)	0/5
caesarean section	362/2343 (15.5%)	250/1613 (15.5%)	12/59 (20.3%)	22/139 (5.8%)	1/5
Unfavorable obstetric outcomes: preterm or low birth weight, n (%)	325/3634 (8.9%)	128/1889 (6.8%)	4/82 (4.9%)	22/196 (11.2%)	0/7
Baseline level anxiety (STAI), mean (SD) ^b^	33 (9.20)	30 (5.97)	57 (8.90)	48 (5.07)	39 (2.62)
Postpartum level anxiety (STAI), mean (SD) ^c^	32 (10.27)	31 (8.91)	48 (14.86)	40 (11.47)	37 (4.19)
Baseline level depression (EPDS), mean (SD) ^d^	4 (3.77)	4 (2.67)	16 (2.95)	8 (2.61)	13 (0.38)
Postpartum level depression (EPDS), mean (SD) ^e^	5 (4.06)	4 (3.46)	12 (5.58)	7 (4.88)	7 (2.50)

SD, standard deviation; STAI, Spielberger State-Trait Anxiety Inventory (min–max 20–80); EPDS, Edinburgh Postnatal Depression Scale (min–max 0–30). * Due to the low number of women in this group, no percentages were calculated.

**Table 2:** Associations of postpartum levels of anxiety and depression with antenatal symptoms, specific life events, and delivery complications. *N* = 3842.

Variables	Postpartum Anxiety Symptoms (*N* = 283)		Postpartum Depression Symptoms (*N* = 92)	
	B (95% CI)	*p*-Value	B (95% CI)	*p*-Value
Anxiety baseline	5.68 (5.27, 6.09)	<0.001	0.57 (1.81, 2.28)	<0.001
Depression baseline	2.78 (2.16, 3.40)	<0.001	2.47 (2.31, 2.62)	<0.001
Events—pregnancy	0.11 (−0.44, 0.66)	0.702	−0.05 (−0.26, 0.16)	0.610
Events—health and sickness of self or loved ones	1.03 (0.55, 1.52)	<0.001	0.36 (0.18, 0.54)	<0.001
Events—partner relation or conflicts with loved ones	1.73 (1.06, 2.40)	<0.001	0.50 (0.24, 0.76	<0.001
Events—work, finance, or housing problems	1.01 (0.70, 1.32)	<0.001	0.23 (0.10, 0.35)	<0.001
Mode of delivery				
*Spontaneous vaginal delivery vs. vacuum or forceps*	−0.61 (−2.02, 0.80)	0.399	0.48 (−0.07, 1.02)	0.085
*Spontaneous vaginal delivery vs. caesarean section*	0.01 (−1.18, 1.19)	0.992	0.23 (−0.23, 0.68)	0.326
Preterm delivery or small for gestational age	0.90 (−1.02, 2.81)	0.359	0.29 (−0.43, 1.01)	0.434

Multivariable linear regression analyses. Antenatal levels of anxiety and depression were standardized by calculating z-scores. Analyses on symptoms of postpartum anxiety were adjusted for baseline anxiety levels. Analyses on symptoms of postpartum depression were adjusted for baseline depression levels. CI, confidence interval.
